# Effect of Alcohol Use on Injection and Sexual Behavior among People Who Inject Drugs in Tehran, Iran: A Coarsened Exact Matching Approach 

**Published:** 2018-05-19

**Authors:** Mehdi Noroozi, Brandon D.L. Marshall, Alireza Noroozi, Bahram Armoon, Hamid Sharifi, Mostafa Qorbani, Mohammad Abbasi, Mohammad Rafi Bazrafshan

**Affiliations:** ^1^Social Determinants of Health Research Center, University of Social Welfare and Rehabilitation Sciences, Tehran, Iran; ^2^Department of Epidemiology, Brown University School of Public Health, Providence, Rhode Island, USA; ^3^Iranian National Center for Addiction Studies (INCAS), Tehran University of Medical Sciences (TUMS), Tehran, Iran; ^4^Department of Neuroscience and Addiction Studies, School of Advanced Technologies in Medicine (SATiM), Tehran University of Medical Sciences, Tehran, Iran.; ^5^Student Research Committee, School of Health, Shahid Beheshti University of Medical Sciences, Tehran, Iran; ^6^HIV/STI Surveillance Research Center, and WHO Collaborating Center for HIV Surveillance, Institute for Futures Studies in Health, Kerman University of Medical Sciences, Kerman, Iran; ^7^Non-communicable Diseases Research Center, Alborz University of Medical Sciences, Karaj, Iran; ^8^School of Nursing and midwifery, Qom University of Medical Sciences, Qom, Iran; ^9^Department of Nursing, School of Nursing, Larestan University of Medical Sciences, Larestan, Iran

**Keywords:** Alcoholism, Injections, Sexual behavior, Drug users, Iran

## Abstract

**Background:** Many studies have recognized the importance of sexual and injection risk behaviors in HIV transmission among people who inject drugs (PWID). We aimed to examine effect of alcohol use on sexual and injection risky behavior using coarsened exact matching (CEM) approach among PWID in Tehran, Iran.

**Study design:** A cross-sectional study.

**Methods:** This study was conducted from Mar to Aug 2016 in Tehran, Iran. PWID were recruited by a convenience and snowball sampling from five of Drop-in Centers (DICs) in Tehran. We assessed three outcomes of interest, all treated as binary variables, including distributive and receptive sharing and inconsistent condom use with any type of sexual partner. We applied coarsened exact matching (CEM) to estimate the independent effect of alcohol use on injection and sexual risk behaviours. All data analysis was performed using Stata v.11.

**Results:** Overall, 550 PWID (all male) were enrolled. The prevalence of receptive sharing, distributive sharing, and inconsistent condom use was 32%, 15% and 55%, respectively. In the matched samples, last month drinkers were more likely to report receptive sharing (OR=2.12 95% CI: 1.31, 3.81; *P*=0.022), as compared to abstainer participants. Distributive sharing in last 30 d, was also significantly higher among last month drinkers group (OR=2.72 95% CI 1.72, 4.21; *P*=0.011), compared last month abstainers group. Finally, there was a statistically significant relationship between alcohol use and inconsistent condom use (OR=4.21 95% CI: 2.71, 7.52; *P*=0.013).

**Conclusions:** The findings emphasize importance of addressing alcohol use in risk reduction interventions for Iranian PWID with alcohol use.

## Introduction

Many studies have recognized the importance of sexual and injection risk behaviors in HIV transmission among people who inject drugs (PWID)^1^. Moreover, alcohol use has been identified as an important risk factor in HIV transmission through sexual risk behavior (e.g., unprotected sex) and unsafe injection (e.g., needle sharing) among PWID^2-4^. Sexual risk behavior was common among people who concurrently use drugs with alcohol^5,6^, and alcohol consumption is a significant factor predicting risky sexual behavior^4^.

Several international studies have assessed the impact of alcohol consumption on self-reported risk behaviours, including unsafe sexual behaviors among PWID. A direct relationship was found between alcohol consumption and needle sharing among PWID participating in a needle, syringe program (NSP)^7^. Moreover, heavy drinking was associated with unsafe sexual behaviors among male PWID ^8^. In addition, a study in Denver, US showed heavy drinking might heighten the risk of unsafe sexual behaviors in PWID. According to WHO in 2005^9^, the relationship between alcohol use and sexual risk behaviors may be influenced by different drinking patterns and cultural norms that exist across different national setting^10,11^. Therefore, it is important to examine the association between alcohol use and sexual risk behaviors in each national setting^9,12^.

Effect of alcohol use on HIV risk behaviour typically utilize observational study designs and subsequently have methodological limitations (i.e., confounders and selection bias)^13^. Experimental evidence for the effect of alcohol use in the form of a randomized-controlled trial has not been possible because of the logistical and ethical problems^14^. Therefore, there is a need for using novel approaches to examine the impact of alcohol use on sexual and injection risk behaviour. Little is known on the effect of alcohol use on injection and sexual behavior among PWID in developing country especially Iran.

The objective of this study was to examine the effect of alcohol use on sexual and injection risky behaviour using coarsened exact matching (CEM) approach among PWID in Tehran, Iran.

## Methods

### Study sample and procedures

The cross-sectional study was conducted from Mar to Aug 2016 in Tehran, Iran. PWID were enrolled using a convenience and snowball (i.e., using peers to refer participants to the study) sampling from five of Drop-in Centers (DICs) located at south of Tehran. Inclusion criteria were age 18 yr or more, using drugs through injection at least once during the last month, and to be able to provide informed consent to complete the interview.

Trained interviewers administered the questionnaire. The questionnaire included modules on socio-demographic characteristics (age, marital status, education level and employment status), and drug use history (age at initiation of drug use, and years of drug use). Risky injecting behaviors included frequency of injection, receptive syringe sharing (obtaining and using a syringe after being used by someone else), and distributive syringe sharing (giving someone else a syringe after you have used it). All behavioral questions referred to the month prior to completing the interview. We assessed three outcomes of interest, all treated as binary variables, including distributive and receptive sharing and inconsistent condom use with any type of sexual partner.

The receptive sharing variable was derived from a survey question which asked participants: ‘‘In the last 30 d, with how many people did you use a needle after they injected with it?’’ The responses were dichotomized into any receptive sharing in the last month (yes vs. no). The unprotected sex variable was derived from survey questions which asked participants about engaging in sex with a partner without using a condom in the last month (including commercial and casual partners). We calculated the intra-class correlation coefficient (ICC) as a measure of inter-rater reliability. Intra-class correlation coefficient was 0.87. Our primary exposure of interest was whether study participants had alcohol use in the month prior to the interview. We created two groups: exposure (i.e., last month drinker) and control (i.e., last month abstainer) groups. No identifying information was collected from questionnaire respondents.

Verbal and written consent procedures were provided to all participants before the survey. Ethics approval was obtained from Ethics Committee of University of Social Welfare and Rehabilitation (USWR), Tehran, Iran.

### Analysis methods

We applied coarsened exact matching (CEM) to match the groups based on certain covariates and thus made statistically equivalent comparison groups to estimate the independent effect of alcohol use on injection and sexual risk behaviours. The CEM created comparable subgroups based on housing status, income, receiving opioid maintenance treatments (OMTs), education level, access to NSP and methamphetamine use. We chose CEM over other matching techniques, such as propensity score matching, to reduce the need for multiple iterations and re-matching, and to maximize the number of possible matches in our sample^15^.

Using CEM, we allocated every study participant into one of a specified set of strata in which all were exactly matched on a set of coarsened variables. Matched members were then assigned a weight specific to that stratum and representative of the proportion of all members present in that stratum. Then, we calculated a statistical measure called L1 distance. It varies between 0 and 1: values close to zero indicate that the matching is perfect and ensures the comparability of the two groups^16^. We calculated the L1 before and after applying CEM, and we observed that L1 decreased from 0.44 to 0.0005 after coarsened exact matching (indicating minimal imbalance between the two comparison groups). Then, we reported the descriptive statistics for the pooled sample and matched sub-samples. We applied a logistic regression model to estimate the effect alcohol use on risk behaviours. The effects were reported as OR and 95% CI. All data analysis was performed using Stata v.11.

## Results

### Characteristics of study participants

Overall, 550 PWID (all male) were enrolled. Table 1 are presented the characteristics of participants in pooled (unmatched) and matched sub-sample.

**Table 1:** Demographic characteristics and risk behaviours (matched and unmatched samples) among PWID, Tehran, 2016

**Variables**	**Pooled sample**		**Matched sampled**	
	**Number**	**Percent**	**Number**	**Percent**
Age (yr)				
<30	275	50.0	180	45.0
30-39	165	30.0	100	25.0
≥40	110	20.0	120	30.0
Educational level				
Primary or no education	165	30.0	100	25.0
High school	220	40.0	200	50.0
Diploma or higher	165	30.0	100	25.0
Monthly income (US$)				
<150	440	80.0	340	85.0
≥150	110	20.0	60	15.0
Age of first drug injecting (yr)			
<25	314	57.0	260	65.0
≥25	236	43.0	140	35.0
Age of first drug use (yr)				
<25	413	75.0	320	80.0
≥25	137	25.0	80	20.0
Number of injection per day in the past month		
<2	192	34.9	160	40.0
≥2	358	64.1	240	60.0
Receptive sharing in past month	220	40.0	128	32.0
Distributive sharing in past month	121	22.0	60	15.0
Inconsistent condom use in the past month	330	60.0	220	55.0
Alcohol use in past month	248	45.0	200	50.0

The matched sample (n=400) had a mean age ±SD of 33.9±8.9 (min: 18, max: 60) yr. The mean age of first drug injection ±(SD was 26.7 ±12.5 yr. Moreover, 30% of participants had less than a high school education, 60% had more than 2 injections per day, and 80% had monthly income less than 150 US Dollars (USD).

### Behavioraloutcomes of study participants

Overall, 50% of the sample reported alcohol use during month prior to the interview. Table 2 shows alcohol use outcomes in the matched and unmatched study sub-samples.

**Table 2:** Logistic regression model for estimates of effect of alcohol use on injecting and sexually risk behaviours in matched and unmatched samples of PWID, Tehran, 2016

**Behavioural outcomes**	**Unmatched sample OR (CI% 95)**	**Matched sampl e OR (CI%9 5)** ^a^
Receptive sharing in past month		
Past month abstainers	1.00	1.00
Past month drinkers	1.71 (1.31, 5.72)	2.13 (1.33, 3.81)
Distributive sharing in past month		
Past month abstainers	1.00	1.00
Past month drinkers	1.92 (1.22, 3.23)	2.72 (1.72, 4.21)
Inconsistent condom use in past month		
Past month abstainers	1.00	1.00
Past month drinkers	3.71(1.30, 6.86)	4.21(2.71, 7.52)

^a^The matched subsample was made by considering, income, education, opioid maintenance therapy (OMTs), access to NSP and methamphetamine use.

Only matched results are presented here. About 32% of respondents reported “receptive sharing in past month” and 15% reported “distributive sharing in past month”. In addition, 55% of participants reported “inconsistent condom use with any kind of sexual partner” in last 30 days. The mean number of injecting partners with whom the study participants had shared with needle, syringes or cooker was 2.1 (SD=1.35). Table 2 presents injecting and sexually risk behaviours in the pooled and matched sub-samples by alcohol use history in past month. In the matched samples, last month drinkers were more likely to report receptive sharing (OR=2.1 95% CI: 1.3, 3.8; *P* =0.022), as compared to abstainer participants. Distributive sharing in last 30 d, was also significantly higher among last month drinkers group (OR=2.7 95% CI 1.7, 4.2; *P* =0.011), compared last month abstainers group. Finally, there was a statistically significant relationship between alcohol use and inconsistent condom use. Alcohol user had higher odds of inconsistent condom use as compared to last month abstainers (OR=4.2 95% CI: 2.7, 7.5; *P* =0.013).

## Discussion

We aimed to examine effect of alcohol use on sexual and injection risky behavior using coarsened exact matching (CEM) approach among PWID in Tehran, Iran. Our reults suggest that alcohol use was associated with receptive sharing, distributive sharing and increase inconsistent condom use with any kind of sexual partners. Primary drug of PWID in Iran is opioids^17-19^ and data regarding concurrent alcohol use are limited.

Fifty percent of the PWID reported alcohol use during last month, which was higher than last month alcohol use (8.5%) reported in a study among PWID in Tehran and 5 other Iranian cities in 2013^20^. Further studies are needed to explore the factors affecting geographical distribution of concurrent alcohol use among PWID in Iran. Overall, 60% of PWID referring to an NSP in US had history of alcohol use in last month and 28% met criteria for alcohol abuse^7^. This discordance could be explained by legal availability and cultural acceptance of alcohol use in US. Using the matched analysis, we found that last month alcohol use was associated with more receptive sharing in last 30 days. The odds of receptive sharing in PWID of last month drinkers group was 2.1 fold higher than PWID of last month abstainers group, in line with other studies where alcohol use was associated with needle sharing behavior ^7,21,22^. We observed significant effect of last month alcohol use on distributive sharing in last 30 days. In a similar study, alcohol use was associated with distributive sharing of needles/syringes^21^. Our finding indicated that there was association between alcohol use and inconsistent condom use, in consistent with other studies where alcohol use was more likely to engage in unprotected sex^9,23,24^. These observations emphasize the importance of addressing alcohol use in harm reduction programs. Moreover, these findings suggests important information for developing sexual risk reduction interventions for PWID with alcohol use.

There are several limitations to the ﬁnding of our study. First, like any cross-sectional study, we could only report the association of exposure with high-risk behaviors. Second, we did not measure the frequency and quantity of alcohol drinking among our participants. It is possible that frequency and pattern of drinking underlie the associations between alcohol drinking and injection and sexual risk behaviors that we observed. Third, we matched the two groups based on income, education, opioid maintenance treatments (OMTs), access to NSP and methamphetamine use; however, we could not account for other factors.

Despite these limitations, the findings from this study provide important information for developing effective sexual and injection risk reduction interventions for Iranian PWID with alcohol use. There are limited informed educational efforts with regard to alcohol use among PWIDs in Iran. HIV prevention education should be more directly address the HIV risk behaviors that accompany alcohol consumption. These efforts should include prevention and harm-reduction programs, and differentially target drinkers and non-drinkers.

## Conclusion

Alcohol use is associated with sexual and injection risk behaviors among PWID in Tehran. The findings emphasize importance of addressing alcohol use in risk reduction interventions for Iranian PWID with alcohol use. This intervention could be done by integration of alcohol use screening and individualized risk reduction services within current harm reduction services.

## Ethical approval

All procedures performed in studies involving human participants were in accordance with the ethical standards of the institutional and/or national research committee and with the 1964 Helsinki declaration and its later amendments or comparable-ethical standards.

## Acknowledgements

We gratefully thank all staff in the Drop-in Centers (DICs) in Tehran who contributed to recruiting and data collection/ interview. We thank participants for their time and interest in the study.

## Conflict of interest statement

All authors have no conflicts of interest to be declared.

## Funding

This study, funded by Substance Abuse Prevention and Treatment Office (SAPTO) of Mental Health, Social Health and Addiction Department (MeHSHAD) of Ministry of Health and Medical Education, Iran.

## Highlights

- There was a statistically significant relationship between alcohol use and inconsistent condom use.
- Distributive sharing in last 30 days, was also significantly higher among last month drinkers group, compared last month abstainers group.
- The findings emphasize importance of addressing alcohol use in risk reduction interventions for Iranian PWID with alcohol use.
